# Secreted α-Klotho maintains cartilage tissue homeostasis by repressing *NOS2* and *ZIP8-MMP13* catabolic axis

**DOI:** 10.18632/aging.101481

**Published:** 2018-06-19

**Authors:** Paul Chuchana, Anne-Laure Mausset-Bonnefont, Marc Mathieu, Francisco Espinoza, Marisa Teigell, Karine Toupet, Chantal Ripoll, Farida Djouad, Danièle Noel, Christian Jorgensen, Jean-Marc Brondello

**Affiliations:** 1 IRMB, INSERM, Montpellier University, Montpellier, France; 2 INSERM U1051, Montpellier, France; 3 CHU Montpellier, Montpellier, France

**Keywords:** α-Klotho, hormone, homeostasis, cartilage, aging

## Abstract

Progressive loss of tissue homeostasis is a hallmark of numerous age-related pathologies, including osteoarthritis (OA). Accumulation of senescent chondrocytes in joints contributes to the age-dependent cartilage loss of functions through the production of hypertrophy-associated catabolic matrix-remodeling enzymes and pro-inflammatory cytokines. Here, we evaluated the effects of the secreted variant of the anti-aging hormone α-Klotho on cartilage homeostasis during both cartilage formation and OA development. First, we found that α-Klotho expression was detected during mouse limb development, and transiently expressed during *in vitro* chondrogenic differentiation of bone marrow-derived mesenchymal stem cells. Genome-wide gene array analysis of chondrocytes from OA patients revealed that incubation with recombinant secreted α-Klotho repressed expression of the NOS2 and ZIP8/MMP13 catabolic remodeling axis. Accordingly, α-Klotho expression was reduced in chronically IL1β-treated chondrocytes and in cartilage of an OA mouse model. Finally, *in vivo* intra-articular secreted α-Kotho gene transfer delays cartilage degradation in the OA mouse model. Altogether, our results reveal a new tissue homeostatic function for this anti-aging hormone in protecting against OA onset and progression.

## Introduction

Tissue homeostasis is ensured through self-repair of specialized cells and their replacements via differentiation of tissue-specific adult stem cells [1,2]. During aging, this equilibrium is gradually lost [2] through the expression of cellular senescence markers in tissues and the establishment of a senescence-associated secretory phenotype (SASP), that includes inflammatory and catabolic factors [2].

Osteoarthritis (OA) is the most common age-related osteoarticular disease characterized by the progressive loss of cartilage homeostasis, synovial activation and sub-chondral bone remodeling [3,4]. The molecular mechanisms responsible for OA onset have just started to be deciphered [4]. Repetitive mechanical stress and the resulting synovial inflammation trigger the acquisition of a p16^INK4A^–dependent senescence phenotype by articular chondrocytes [5] that resembles to their terminal differentiation program during endochondral ossification [5,6]. For instance, the production of many deleterious factors that alters the balance between anabolic and catabolic articular functions is a central event. Particularly, IL-1β plays a key role in OA onset by inducing in articular chondrocytes: (1) DNA damage accumulation through nitric oxide synthase 2 (NOS2)-dependent nitric oxide production [7], (2) the expression of senescence marker p16^INK4A^ [6] (3) the onset of the hypoxia-inducible factor 2 (HIF2)-dependent terminal phase of endochondral ossification [8,9], and (4) the activation of the catabolic axis formed by the zinc importer ZIP8, metal-regulatory transcription factor-1 (MTF1) and the matrix metalloproteinase-13 (MMP13) [10].

Besides aging, genetic predisposition also favors OA development [11]. For instance, two single nucleotide polymorphisms in *α-KL*, the gene encoding the anti-geronic hormone α-Klotho, predispose to OA onset [12–14]. The human *α-KL* gene encodes four proteins: full length α-Klotho, two soluble variants, and one secreted form. Secreted α-Klotho results from alternative splicing of exon 3 and contains a N-terminal signal peptide and only one glycosyl hydrolase (KL) domain (KL1) with trophic factor-binding properties and low syalidase activity (for review [12],). The full-length transmembrane form contains two KL domains (KL1 and KL2) and acts as co-receptor of growth factors (such as FGF23 and VEGF) [12,15]. This membrane-bound form can also be cleaved by a disintegrin and metalloprotease (ADAM) cell surface proteases to form the two soluble KL1-KL2 and KL1 forms [16] that control the Ca2^+^/K^+^ reabsorption activity of transient receptor potential cation channel subfamily V member 5 (TRPV5) in kidney epithelial cells (for review [12],). Although the secreted and the short soluble form of α-Klotho are very similar (both harbor only the KL1 domain), the former has in addition a C-terminal extension of few amino acids [17].

The serum level of secreted α-Klotho decreases with aging in mice and humans [18], and secreted α-Klotho can inhibit TGFβ1-induced fibrosis and signaling pathways induced by Wnt/β-catenin and Insulin-like Growth factor-1 (IGF-1) (for review [12],). Finally, mice in which *α-KL* was knocked out show growth retardation, osteoporosis, ectopic calcification in soft tissue, premature tissue aging and death at 9 weeks of age [12,19].

In the present study, we evaluated the link between secreted α-Klotho expression, cartilage homeostasis and OA onset/progression. We determined the expression level of α-Klotho (all variants) during mouse limb development, and that of the secreted form during *in vitro* differentiation of bone marrow-derived mesenchymal stem cell. To determine the role of this alternative-spliced form for α-Klotho in cartilage homeostasis, we compared the expression profile of primary human chondrocytes from OA patients incubated with recombinant secreted α-Klotho or after *α-KL* silencing using a RNA interference approach. We identified a set of genes encoding catabolic factors that are repressed upon incubation with secreted α-Klotho and that are known to control OA-dependent tissue degeneration and hypertrophy. Using *in vitro* and *in vivo* OA models, we determined the expression level of secreted α-Klotho during OA and tested whether intra-articular *α-KL* gene transfer has chondroprotective effects.

## RESULTS

### α-Klotho is expressed during cartilage formation in mouse embryos

**Figure 1:**
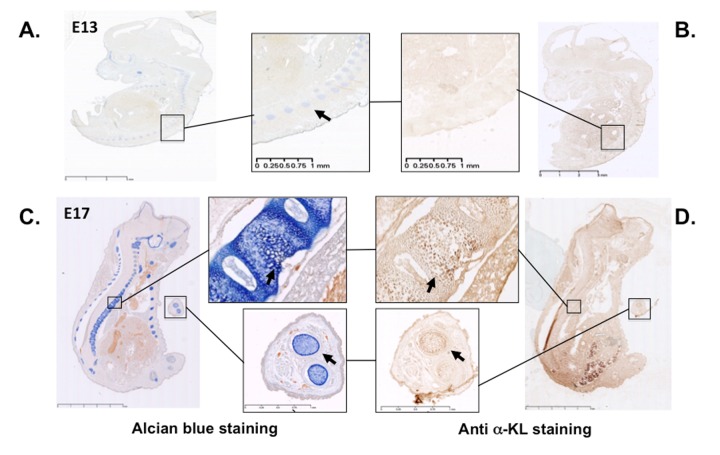
**α-Klotho expression in mouse embryos during cartilage formation**. Serial sections of E13 and E17 mouse embryos were stained with Alcian blue to detect cartilage (**A** and **C**) or with an anti- pan α-Klotho antibody (**B** and **D**). Arrows are showing cartilaginous tissues expressing also α-Klotho.

In mice, α-Klotho expression is detected in proliferative chondrocytes within the growth plate during endochondral ossification [20], as expected on the basis of the growth retardation phenotype described in *α-KL* knock-out mice [19]. These findings prompted us to assess α-Klotho expression during limb development using immunostaining approach with an antibody against all α-Klotho variants. Incubation of cryo-sections of mouse embryos at day 13 and 17 of embryonic development (E13 and E17) with this antibody showed that α-Klotho expression was barely detectable at E13, during formation of the cartilage primordia (Figure 1A-B). In contrast, it was clearly detected at E17 within cartilage rich-tissues, such as the spine and digits, as revealed by co-staining with Alcian blue (Figure 1C-D).

### Secreted *α-KL* is expressed during *in vitro* chondrogenesis

**Figure 2:**
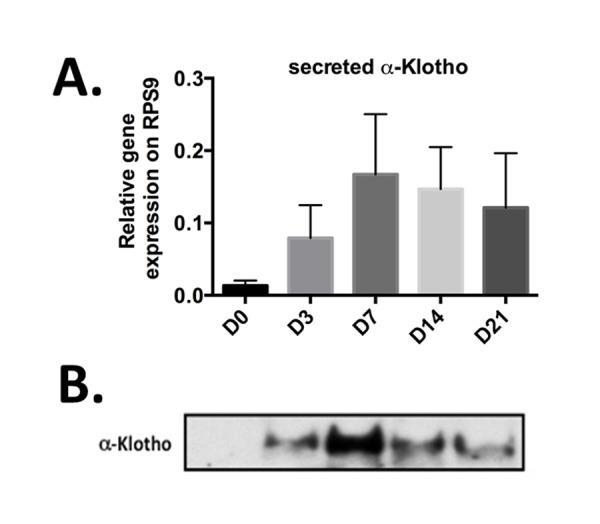
**Secreted α-Klotho expression during *in vitro* chondrogenesis.** TGFβ3-driven chondrogenic differentiation of BM-MSCs (osteochondral progenitors) in micropellet culture (n=3). Expression analysis of secreted *α-KL* (**A**) by RT-qPCR (gene expression level relative to that of *RPS9*) at different time points during BM-MSC differentiation into chondrocytes. (**B**) α-Klotho protein expression detectable below 65kDa marker by western blotting in conditioned medium from BM-MSCs in micromass culture at the different time points during chondrogenesis. Data are represented as mean -/+ SEM.

To develop potential innovative articular therapeutic approaches, we next wanted to specifically evaluate the expression level of secreted α-Klotho during cartilage formation. To do so, we ordered primers that allowed the specific detection of only this form by RT-qPCR [17,21]. We used bone marrow-derived mesenchymal stem cells (BM-MSC) in micropellet culture with TGFβ3 as *in vitro* chondrogenic progenitors to recapitulate *in vitro* the different stages of chondrogenic differentiation that occur during cartilage formation and *in vivo* endochondral ossification [22]. We exploited our previously published chondrogenic kinetic experiments, where *Col2B* and *MMP13* mRNA expression levels, which encode for markers of mature chondrocytes and terminally hypertrophic differentiated chondrocytes respectively, reached their peak of expression at day 21 [6]. We monitored on the same samples, the expression of secreted *α-KL* by Rt-qPCR and western-blot during 21 days (Figure 2). At day 0, secreted *α-KL* was barely detectable in proliferating BM-MSCs (Figure 2 A-B). Upon TGFβ3 addition, the expression of secreted *α-KL* increased and peaked at day 7 and then progressively decreased until day 21 (Figure 2B), when *Col2B* and *MMP13* reached their maximum [6]. The temporal changes in the expression of secreted α-Klotho were confirmed also by western blot analysis of conditioned medium from BM-MSCs in micropellet culture (Figure 2B). In summary, expression of α-Klotho is up regulated during early *in vitro* chondrocyte differentiation and *in vivo* during cartilage formation.

### Secreted α-Klotho regulates the expression of major homeostatic pathways in human articular chondrocytes

**Figure 3:**
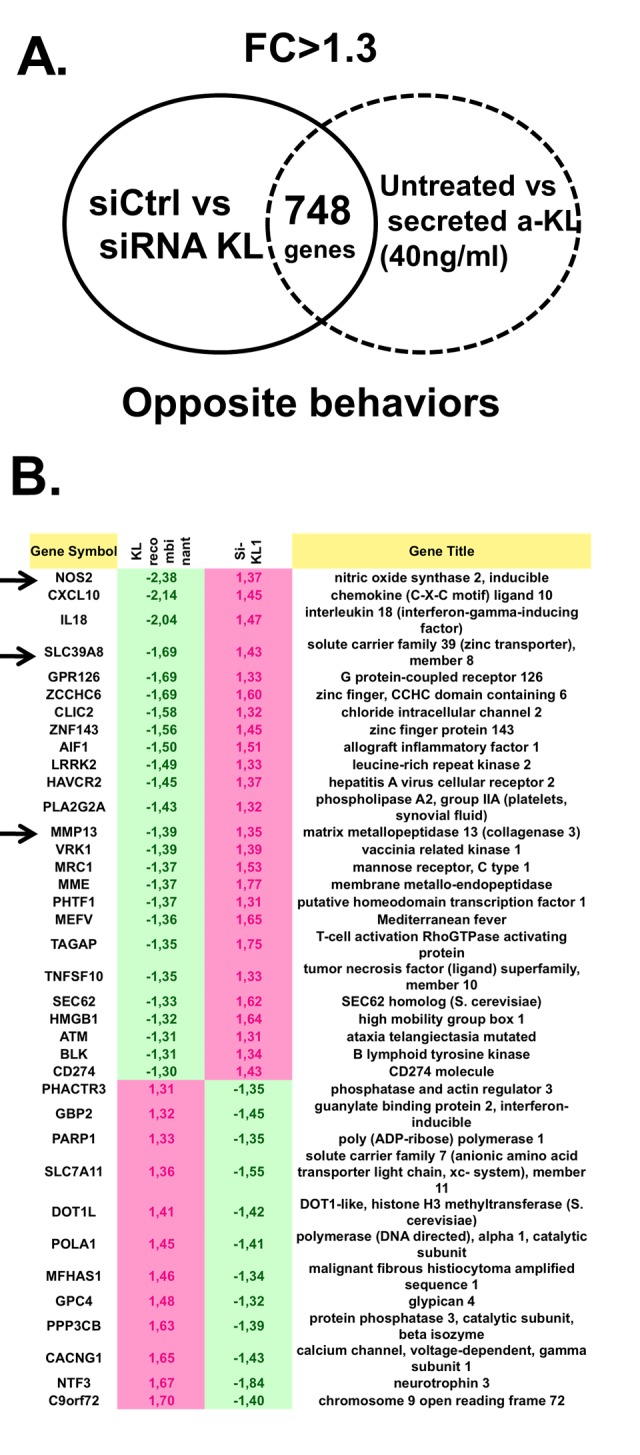
**Identification of genes regulated by secreted α-Klotho in human chondrocytes**. (**A**) Genome-wide microarray analysis of human primary chondrocytes incubated or not with recombinant secreted α-KL or after siRNA-mediated *α-KL* silencing identified 748 genes with opposite behavior in these two experimental conditions. FC, fold change. (**B**) List of the 38 genes (among the common 748 differentially expressed genes) that are involved in osteoarticular diseases according to the Ingenuity® software.

To determine the specific role played by secreted α-Klotho in articular chondrocytes, we used primary human chondrocytes isolated from cartilage of patients after arthroplasty surgery. We incubated these isolated cells or not with recombinant secreted α-Klotho, purified as previously described [21], or we transfected them with an irrelevant siRNA or with a siRNA targeting all *α-KL* variants. After 7 days of culture, we isolated total RNA from cells in these different culture conditions and performed a genome-wide microarray analysis to identify genes the expression of which was modulated by recombinant secreted α-Klotho and by *α-KL* silencing compared with the respective controls. To strictly select gene transcripts modulated by secreted α-Klotho, we choose only genes for which the expression was affected in the opposite direction by α-Klotho addition and *α-KL* silencing (Figure 3A). We then used the Ingenuity® software to identify among these 748 differentially expressed genes, known factors with osteoarticular homeostatic functions. We could restrict the list to 38 genes that were positively or negatively regulated upon secreted α-Klotho addition or α-KL silencing (Figure 3B). Among the genes upregulated upon *α-KL* silencing and repressed by incubation with secreted α-Klotho, we found *NOS2* and *MMP13,* two major cartilage homeostatic players, and also SLC39A8 that encodes the Zin2+ transporter ZIP8 (Figure 3B, arrows). NOS2 and the ZIP8-MMP13 axis are central actors in OA-dependent cartilage alteration. Moreover, NOS2 and MMP13 are involved also in endochondral ossification [8,10,23]. Interestingly, our data also revealed that secreted a-Klotho modulate the expression of senescence regulatory genes such as HMGB1, recently associated with OA onset [5], ATM and DOT1L, an epigenetic regulator found in GWAS analysis from OA patients [24]. Altogether, these suggest that α-Klotho might have a role in cartilage homeostasis by preventing OA onset/progression.

### α-Klotho expression is repressed in *in vitro* human and *in vivo* mouse models of OA

**Figure 4:**
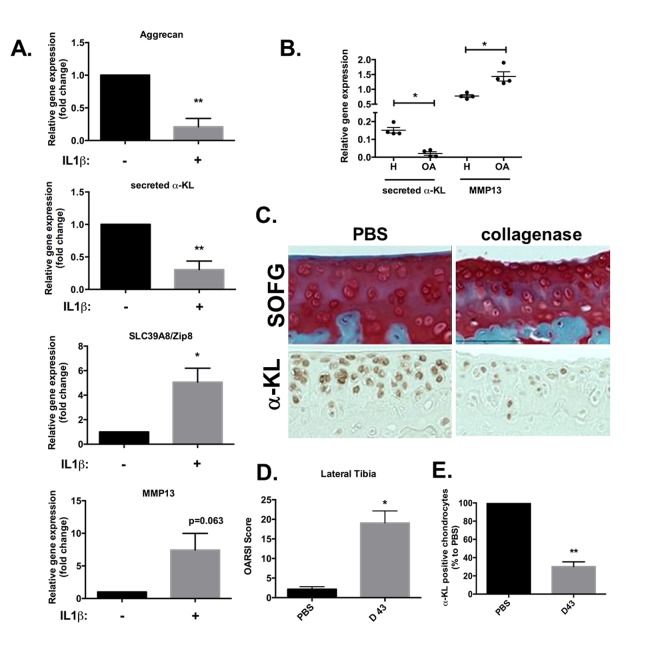
**α-Klotho expression is reduced in osteoarthrosis models.** (**A**) *ACAN*, secreted *α-KL*, *SLC39A8* and *MMP13* mRNA expression analysis by RT-qPCR of human chondrocytes incubated or not with IL-1β for 10 days. (**B**) Secreted *α-KL* and *MMP13* expression levels in human OA (n=4) versus healthy cartilage (n=4). (**C**) α-Klotho expression by immunostaining (bottom panel) in joint knee cartilage at day 43 after intra-articular injection of PBS (n=5 mice) or collagenase (contralateral knee). Top panels show Safranin-O Fast-Green (SOFG) staining of the same joints. (**C**) OARSI scoring of cartilage degradation and (**D**) quantification of α-Klotho-positive chondrocytes in joints at day 43 after intra-articular injection of PBS or collagenase. Data are represented as mean -/+ SEM. *=p<0.05; **=p<0.01.

Therefore, we determined whether the expression level of secreted α-Klotho was affected during OA. To this aim, we first used an *in vitro* mimetic model of OA based on human primary chondrocytes chronically exposed to IL-1β. After 10 days of incubation with IL-1β, the expression levels of secreted *α-KL* and of *ACAN,* a cartilage matrix encoding protein*,* were significantly reduced, while the expression of *SLC39A8* and *MMP13* catabolic axis was increased as expected (Figure 4A) [10]. This showed an inverse correlation between the expression of secreted *α-KL* and this major OA-promoting catabolic axis and suggested that *α-KL* repression is a driving force in OA onset. We confirmed this finding on human cartilage, by analyzing through RT-qPCR, the expression level of secreted α-Klotho and MMP13 (Figure 4B) in cartilage samples from OA patients and healthy controls that we used in a previous study [6]. To finally *in vivo* validate these results on an OA experimental model, we relied on intra-articular injections of collagenase in mice (Figure 4C-E) [25]. In this model, both inflammation and mechanical stress contribute to cartilage loss of function [26]. At day 43 after collagenase injection in the knee joint, mice were sacrificed and limbs isolated to determine the level of cartilage degradation, compared with the contralateral joint that received an injection of phosphate buffer saline (PBS), using the international scoring (OARSI) method [26,27] after staining with Safranin-O Fast-Green (Figure 4C). The OARSI score for the lateral tibio-femoral component of the knee joint was significantly increased in the treated compared with the untreated (PBS) contralateral joint, thus confirming OA induction in our model (Figure 4D). Moreover, analysis of α-Klotho expression in these joints (as described in Figure 1) showed that the number of α-Klotho-positive chondrocytes was significantly decreased in collagenase-treated knees compared with the contralateral PBS-treated joints (Figure 4C and E). Altogether, these *in vivo* findings validated the results obtained in human chondrocytes and cartilages strongly suggesting that reduced level of secreted α-Klotho is associated with chondrocyte loss of function in OA.

### Intra-articular secreted *α-KL* gene transfer reduces disease severity in an OA mouse model

**Figure 5:**
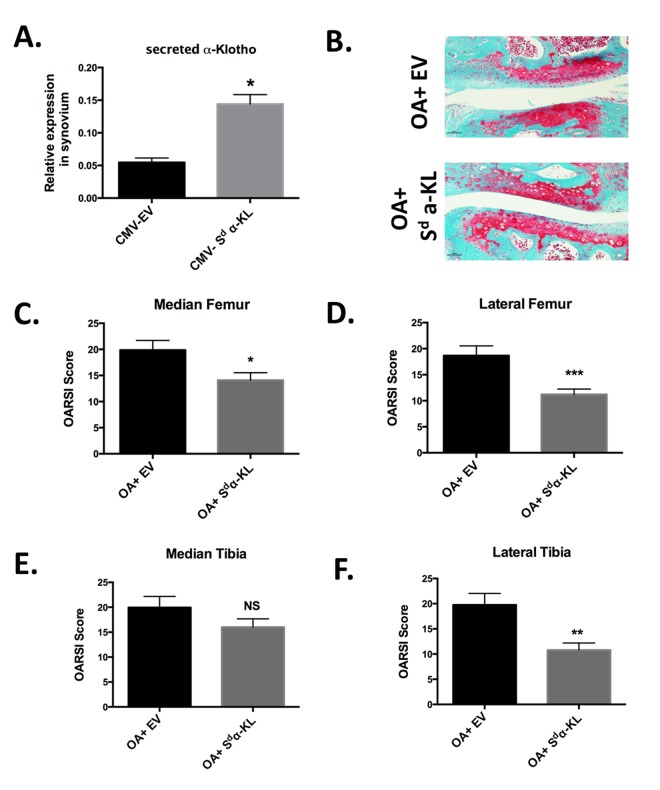
**Effect of intra-articular secreted α-KL gene transfer in an experimental murine OA model.** (**A**) Secreted *α-KL* gene expression in mice synovium after electrotransfer of empty vector (CMV-EV) or CMV-S^d^-α-KL expressing vector. (**B**) Representative Safranin-O Fast-Green staining of knee joints from OA mice treated with empty vector (OA+EV) or CMV-S^d^-α-KL expressing vector (OA+S^d^-α-KL). (**C-F**) OARSI scores in the different joint localizations from OA mice after intra-articular electrotransfer of empty vector (OA+EV; n=15) or CMV-S^d^-α-KL expressing vector (OA+S^d^-α-KL; n=25). Data are represented as mean -/+ SEM. *=p<0.05; **=p<0.01.

As secreted α-Klotho could downregulate three major catabolic actors of OA and α-Klotho-positive chondrocytes were significantly decreased in collagenase-treated knees, we next asked whether *in vivo* intra-articular secreted *α-KL* gene transfer in our mouse model of OA could have chondroprotective effects. First, we tested the efficiency of the gene transfer method by electrotransferring in the knee joint a DNA plasmid to express secreted mouse α-Klotho (CMV-S^d^-α-KL) [17] or the corresponding empty vector (CMV-EV) [27]. Using this method, ectopic expression of the protein of interest is detected mainly in joint synoviocytes [28], which are articular specialized fibroblastic cells that provide nutrients and homeostatic factors to chondrocytes within the knee. Indeed, by RT-qPCR we detected a 2.5-fold increase in secreted *α-KL* expression level using total mRNA isolated from dissected synovial membrane of joints after electroporation of CMV-S^d^-α-KL compared with controls (CMV-EV) (Figure 5A).

We next determined whether ectopic intra-articular expression of secreted α-KL modulated disease severity in the mouse model of OA. To this aim, at day 13 after collagenase injection, we intra-articulary electroporated the CMV-S^d^-α-KL construct (n=25 mice) or the empty vector (n=15 mice) in the knee of each mouse. At day 43, OARSI scoring of knee joints after staining with Safranin-O Fast-Green revealed that the OARSI score, and therefore OA severity, was significantly reduced in the lateral femur, median femur and lateral tibia, but not in the median tibia joint component, of treated mice (OA+ S^d^-α-KL) compared with controls (OA+EV) (Figure 5B-F). Altogether, our results demonstrate the protective effects of secreted α-Klotho during OA.

## DISCUSSION

In OA, loss of the balance between anabolic and catabolic factors that ensures joint tissue homeostasis promotes the establishment of a senescence-associated phenotype. Indeed, articular OA chondrocytes are characterized by the production of deleterious inflammatory, reactive oxygen species and p16^INK4a^-dependent expression of tissue remodeling enzymes [6] leading to cartilage degradation. The restoration of the catabolic/anabolic equilibrium is a target of choice for innovative therapeutic interventions in patients with OA.

Here, we found that the secreted form of the anti-aging factor α-Klotho is a new homeostatic regulator of cartilage integrity. This hormone is locally expressed by articular chondrocytes during early chondrogenesis (Figures 1 and 2), and its level is reduced at the terminal differentiation stage (Figure 2). Moreover, in human primary chondrocytes, secreted α-Klotho regulates negatively the expression of the *NOS2* and *MMP13* genes that encode two major articular catabolic factors (Figure 3). NOS2 and MMP13 are indeed central players in OA onset and during endochondral terminal differentiation within the growth plate [29]. *Nos2*-deficient mice show cartilage developmental defect [30], while NOS2-dependent nitric oxide production triggers *in vitro* and *in vivo* OA phenotypes in chondrocytes [29]. The MMP13 catabolic axis has also this dual role by being important during endochondral ossification and OA development [6,31]. We also found that the level of secreted α-Klotho is already decreased when *MMP13* reach its expression peak at the end of *in vitro* chondrogenesis (Figure 2) [6], as well as in human chondrocytes following IL1β treatment (Figure 4A) and in human OA cartilage compared to healthy donors (Figure 4B). These findings argue for a reverse correlation between expression of *α-KL* and these catabolic factors in physiological and pathological conditions. Finally, α-Klotho level is also reduced in murine articular chondrocytes after collagenase-induced OA (Figure 4B). Conversely, ectopic expression of secreted *α-KL* in joints reduces collagenase-induced cartilage degeneration (Figure 5), thus revealing its chondroprotective function. Importantly, our findings cannot rule out a role or even a lack of role(s) for other forms of α-Klotho in cartilage homeostasis as recently published for FGF23/membrane-bound α-Klotho axis [32].

It is not known how secreted α-Klotho can trigger such effects on cartilage. This hormone has no known receptor on the cell surface and its mode of action seems to depend on its ability to bind to and modify glycoproteins (for review [12]). By this way, secreted α-Klotho can directly inhibit several signaling molecules involved in various processes, such as TGFβ1-dependent fibrosis [33], insulin-like growth factor (IGF-1)-induced oxidative stress [34] and Wnt/β-catenin-dependent gene expression [33]. Remarkably, all these three signaling factors have important functions in cartilage homeostasis. Variation in TGFβ1 level indeed controls endochondral ossification and its overexpression can induce OA in mice [35]. IGF-1 regulates articular chondrocyte proliferation and anabolic/catabolic functions [36], and the canonical Wnt signaling cascade promotes expression of matrix-remodeling enzymes by chondrocytes during OA progression [37].

TGFβ1, Wnt/β-catenin-dependent signaling and IGF-1 can also be senescence-promoting factors [38,39] whereas articular OA chondrocytes and OA synovium were recently proven to harbor cellular senescence features during OA onset [5]. Remarkably α-Klotho has been shown to prevent cellular senescence onset in fibroblasts [15], endothelial [40] and epithelial kidney cells [41] meanwhile α-Klotho improves stem cell proliferation and cell survival [42]. Therefore, we could hypothesize that, in healthy joints, the local production of secreted α-KL by chondrocytes modulates the damaging activity of the mechanical loading stress that can drive production of OA-/hypertrophic-associated and pro-senescent factors, exemplified by TGFβ1. With aging, such repeated stresses in articular joints might result in the progressive reduction of secreted α-Klotho level, thus increasing for instance TGFβ1 activity to drive cellular senescence and OA onset. Thus, restoring the articular level of secreted α-Klotho in OA joints could help to reduce cartilage degeneration as shown in Figure 5 through blockade of these deleterious factors.

We demonstrate here for the first time that anti-geronic secreted α-Klotho has a chondroprotective role on articular cartilage and links its repression to OA onset. Recently, heterochronic parabiosis approaches whereby young mice share their blood circulation with old mice allowed the identification of other longevity factors, such as growth differentiation factor-11 (GDF11) (review in [43]). The circulating blood level of both secreted α-Klotho and GDF11 is reduced in elderly and aged mice [43]. Remarkably, systemic delivery of GDF11 in old mice can improve cerebral tissue functions [43] like secreted α-KL does [44]. Thus, through their systemic hormonal action, these two factors broadly control tissue homeostasis by either reducing the levels of oxidative stress, preventing cellular senescence, controlling ectopic tissue calcification, but also by improving cognitive, renal, muscular, cardiac or articular functions ([43,44] and this work). Altogether these bring new therapeutic perspectives based on the systemic or local delivery of such longevity factors to treat age-dependent pathologies.

## MATERIALS AND METHODS

### Cell types and cell culture conditions

Human primary chondrocytes and human primary BM-MSCs were isolated from cartilage and tibial subchondral bone of patients with OA undergoing knee arthroplasty, after informed written consent by the patients and approval by the local and national Ethical Committee “Cellule de bioéthique de la direction générale pour la recherche et innovation, Ministère de l’Enseignement Supérieur et de la Recherche (registration number: DC-2009-1052)” as described in [22]. BM-MSCs were used at passage 3 to 6 and were positive for CD44, CD73, CD90 and CD105, and negative for CD14, CD34 and CD45, as previously described [11]. Primary chondrocytes were cultured at passage 0 and 1 in Dulbecco’s modified Eagle medium (DMEM) containing 10% fetal calf serum. 5x10^5^ confluent chondrocytes were maintained in DMEM supplemented with 0.1μM dexamethasone (Sigma, USA), 1mM sodium pyruvate (Invitrogen, UK), 0.17mM ascorbic acid (Sigma, USA), 0.35mM proline (Sigma, USA), 1% insulin-transferrin-selenic acid (ITS) (Lonza, CH), 2mM L-glutamine (Lonza, CH), 100U/ml penicillin and 100μg/ml streptomycin (Lonza, CH), as described [23]. Human recombinant secreted α-Klotho was purified as previously described from transfected NIH3T3 cells [18]. Confluent human primary chondrocytes were incubated or not with 40ng/ml recombinant secreted α-Klotho for 7 days (refreshed every day) as described [45], or with 10ng/ml recombinant human IL-1β (Bio-Techne, GER) for 10 days.

### *In vitro* differentiation of BM-MSCs into chondrocytes

Chondrogenic differentiation of BM-MSCs was induced by 21-day culture in micropellets [12]. Briefly, 2.5 x 10^5^ BM-MSCs were pelleted by centrifugation in 15ml conical tubes and cultured in chondrogenic medium (DMEM supplemented with 0.1μM dexamethasone, 0.17mM ascorbic acid and 1% ITS) with TGFβ3 (Bio-Techne, GER) [12]. Chondrogenic differentiation was monitored by assessing the expression of chondrogenic markers by RT-qPCR using previously published primers [6]. The expression of secreted α-Klotho was evaluated by western blotting using 20μg of TCA-precipitated total proteins from 2 ml of conditioned medium from BM-MSC cultures at the indicated times during differentiation [46]. Proteins were separated on 10% SDS-PAGE, transferred on PVDF membranes, saturated with 5% fat-milk in PBS and incubated with a rabbit polyclonal anti-αKlotho antibody (1:200; Abcam 75023) overnight. Antibody binding was revealed using a goat anti-rabbit antibody coupled to HRP and ECL, as published ([6].

### Gene expression analysis and vector purification

Total RNA from cultured cells or mouse synovial tissues was extracted with the RNeasy Kit (Qiagen, USA) according to the manufacturer’s instructions. 250 ng of total RNA was reverse transcribed using the M-MLV Reverse Transcriptase Kit according to the manufacturer’s instructions (Thermo Fisher, UK) and using 10µM random hexamers (Thermo Fisher, UK), 1mM dNTP mix, 10mM DTT, 5X RT buffer, 50 units M-MLV Reverse Transcriptase. Complementary DNA (cDNA) was then mixed with the Sybr Green Master Mix (Roche, CH) and specific primers. The published sequences of human and mouse secreted *α-KL* [17] were used. The primer sequences for *NOS2*, *SLC39A8*, and *MMP13* were previously described [10]. Gene expression analysis was performed as previously described using the 2-dCT method and *RPS9* expression level as internal control for human and mouse samples [6].

The PCNA-neo vector (CM-EV) and PCNA-neo-secreted α-KL expressing plasmid (CMV-S^d^-α-KL) used in our *in vivo* experiments were previously described [17]. They were purified from ampicillin-resistant *E. coli* colonies using the Endotoxin-Free Plasmid DNA Isolation Kit (Qiagen, USA) according to the manufacturer’s instructions. The intra-articular expression level of ectopic secreted *α-KL* was verified using total RNA from dissected synovial tissue.

### RNA interference and genome-wide microarray analysis

Total RNA was extracted with the RNeasy Isolation Kit (Qiagen, USA) from 5x10^5^ primary chondrocytes from three different patients with OA incubated or not with recombinant human α-Klotho (40ng/ml) for 7 days. In parallel, total RNA was obtained also from the same three independent human primary chondrocyte cultures transfected twice (at day 1 and 2) with 20nM of control siRNA or the previously described siRNA targeting all *α-KL* variants [47] using Oligofectamine according to the manufacturer’s protocol (Thermo Fisher Scientific, UK). Labeling and hybridization to the HTA-2_0 Human Transcriptome Array 2.0 (Affymetrix, UK) were performed using 200 ng of total RNA according to the manufacturer’s protocol. Raw data were normalized and additional data analysis was performed as described previously [25]. Gene array data are available at the NIH library under the accession number GSE80285.

### Collagenase-induced OA and articular electroporation

Two-month-old C57BL6 male mice were housed and cared for according to the European Directive 2010/63/EU. The experimental protocol was approved by the “Regional ethical committee on animal experimentation” (approval CEEA-LR-10042). At day 0 and day 2, 1U/ml collagenase (Sigma-Aldrich (USA) was injected intra-articularly as previously described [48]. At day 13 post-injection, 10μg of each plasmid was electro-transferred intra-articularly, as previously described [28]. At day 43 post-injection, animals were sacrificed and their limb joints isolated for cartilage analysis.

### Histology, immunohistochemistry and antibodies

C57BL6 mouse embryo cryo-sections were obtained from the RHEM collection (samples were collected according to the European Directive 2010/63/EU). This collection was approved by the regional ethics committee on animal experimentation (approval CEEA-LR-1194). For analysis of the OA mouse model, joints from PBS-treated or collagenase-treated animals were fixed in 4% paraformaldehyde at 4°C for 48h, washed in PBS and then processed for routine histology. Knees were decalcified in 14% EDTA/PBS for three weeks and then paraffin-embedded. Tissue sections (5 µm) were rehydrated through a gradient of ethanol and xylene. Sections were then stained with Safranin-O Fast-Green solution to evaluate cartilage degradation using the OARSI scoring system, or processed for α-Klotho detection with specific antibodies. For this, embryo cryo-sections and paraffin-embedded tissue sections were first incubated at room temperature (RT) with 10μl of pepsin for 20min for epitope retrieval, and then with 1% H_2_O_2_ at RT for 20min to block endogenous peroxidases. Sections were then pre-incubated with PBS/10% goat serum/0.1% Triton X-100 at RT for 30min. Endogenous biotin was blocked with the Streptavidin/Biotin Blocking Kit (Vector Laboratories, SP-2002) for 30min. Then, sections were incubated with the primary anti-α-Klotho polyclonal rabbit antibody (1:200; Abcam 75023) at 4°C overnight, followed by the secondary biotinylated antibody (1:200; Vectastain Elite ABC-HRP Kit, Vector Laboratories, PK6100) at RT for 1 hour. Finally, the Avidin/Biotinylated enzyme complex (ABC Vectastain Elite ABC-HRP Kit, Vector Laboratories, PK6100) was added at RT for 30min. Antibody binding was revealed with the DAB D-4146 kit revelation (Sigma-Aldrich, USA) according to the manufacturer’s instructions.

### Statistical analysis

The unpaired Mann–Whitney test was used to compare the OARSI scores between treated versus untreated mice and human cartilage samples from OA versus healthy donors. Human chondrocyte experiments and cell counting were performed using at least three independent samples. Comparisons of two conditions for the same samples were performed using the paired *t*-test. All statistical analyses were performed with GraphPad Prism (San Diego, USA). P values <0.05 were considered significant.
